# “Gilead is within you”: A Theocratic Narrative Setting of Cultural memory in Atwood’s *The Testaments*

**DOI:** 10.12688/f1000research.132487.2

**Published:** 2024-05-28

**Authors:** Manshi Yadav, Palak Arora, Mandvi Singh

**Affiliations:** 1 Banasthali University, Vanasthali Rd, Aliyabad, Jaipur, Rajasthan, 304022, India; 2 Amity Education Valley, Manesar, Panchgaon, Amity University, Gurugram, Uttar Pradesh, 122412, India; 3 Banasthali Vidyapith, Rajasthan, India

**Keywords:** Atwood, memory, culture, literature, Dystopian

## Abstract

This article attempts to explore the synthetic relationship between group identities and their effects on cultural memory in Margaret Atwood’s The Testaments. It will investigate how Atwood’s dystopian fiction conspicuously weaves threads of cultural identity in the totalitarian society. While examining the work, the researchers found three forms of Memory Figures channelizing society: Architecture and time, Identical groupings, and Language. With significant attention to the prequel, The Handmaid’s Tale, this article will consider The Testaments for the dissection of hard-wired cultural memory formation. This article focuses on how a politically unsettled society of Gilead, through its Memory Figures, inscribes a cultural narrative while instructing individual memories. Atwood’s usage of these Memory Figures to achieve a theocratic regime has been scrutinized in this article. The Architecture of Gilead was given an intrusive wall of glass, where the symbol of eyes prevails, to externalize the surveillance of God on each one of its citizens, thereby forming a cultural memory of subjectivity. To make the citizens identify with groups, this theocratic regime specifies roles to citizens according to their rank for men and according to reproductive efficiency for women. Keeping in view the relevance of language in forming cultural memory, Atwood prescribed a set of greetings such as ‘under his eyes,’ and ‘praise be’ to make profound imprints on the individual memory of citizens, which has been discussed in the study. These three Memory Figures simultaneously form collective memory imprints on the citizens of Gilead, which in time became inherent cultural memories. This article explores how, by politicizing the issue of cultural memory in The Testaments, Atwood taps into the agency of people and establishes language control (communicative) to form the cultural memory narrative.

## Introduction

*The Testaments* (2019) is a sequel to the novel *The Handmaid’s Tale* (1985), a futuristic dystopian novel written by Margaret Atwood. Henceforth, the events in the novel follow on fifteen years from the prequel and set the patriarchal dystopian world in motion. Further subjugating, Agnes and Daisy the daughters of Offred, the protagonist of *The Handmaid’s Tale*, are toiling hard to find their way in Gilead. Atwood, in her creation of this dystopian world probes the miscommunicated excerpts of the Bible such as *‘let the woman learn in silence with all subjection’,* which created the foundations for Gilead ( Atwood, 1996, p. 229). Daisy aka Baby Nicole the star child of Gilead is raised in Canada far from the pseudo religious renditions of Gilead. She came out as an independent thinker since her memory was not tarnished with the cultural probes of religion. On the other hand, Agnes, her half sister turns out to be a total proto Gileadian woman with a subjugated brain and limited religious knowledge. The narrative of *The Testaments* revolves around how these two women, with the help of Aunt Lydia and Mayday, shift the religious autonomy and fight back the cultural imprints of Gilead. Weaving an intricate fabric of subjective cultural memory for its citizens, through the media and memory figures lens defined by Assmanns, in this article we would like to draw focus on the process of cultural memory formation in Atwoods’ theocratic regime. We argue that Atwood highlights three media of cultural memory during her course of writing *The Testaments* i.e., Architecture, Identical Grouping and Language.

## Literature Review

Memory is ever prevailing and quintessentially ambivalent in all human beings. Philosophical interests in memory can be dated back to antiquity and have remained of interest. It formally emerged as a field of enquiry after French philosopher Maurice Halbwachs published his defining work *Social Framework of Memory* in 1925. Ever since, Memory Studies became an intriguing point of inquiry for philosophers, psychologists, sociologists and theologists, and became a transdisciplinary field ( Olick & Robbins, 1998). Being a major preoccupation for social thinkers, Memory Studies evolved as a field from individual and intellectual enquiry to a socially constructed phenomena i.e., through physiology and psychology to social culturology ( Quan, 2019). To untangle the wires of memory being a transient centerless paradigm, it’s important to trace its origins from inception. Maurice Halbwachs, the pioneer figure in the paradigm of “collective memory” when introduced the term, alludes *‘it is in society that people normally acquire their memories. It is also in society that they recall, recognize, and localize their memories’* ( Halbwachs, 1992, p. 38). After Halbwachs’ discourse, historians like Amos Funkenstein oppose the tenets of collective memory altogether, asserting *‘consciousness and memory can only be realized by an individual who acts, is aware, and remembers. Just as a nation cannot eat or dance, neither can it speak or remember. Remembering is a mental act, and therefore it is absolutely and completely personal.’* ( Funkenstein, 1989, p. 6). The idea of memory as a construct of collective remembrance has been toiled over by psychologists and anthropologists, which in the end came out to be a favorably progressive argument like the one propounded by Lev Vygotsky *‘a mind cannot be independent of culture’* ( Karuny, 2022) *.* Shaping the discourse of Collective Memory Studies in a historiographic mould, French historian Pierre Nora in his seminal work *Les Lieux de mémoir* (1984) dispelled all lingering doubts regarding the theoretical status of Halbwachs’ oral memory ( Assmann, 2008b) by coining the concept of *‘place of memory’* to designate concrete places where collective memories can crystallize their existence, hence making way for architecture in collective memory studies ( Nora, 2011). Since cultural memory needs a space to materialize its existence in time, therefore the study of architecture becomes important in the field of Memory Studies.

Progressing through these interdisciplinary tenets of the theory, Jan Assmann, a German Egyptologist wrote a landmark work *Cultural Memory and Early Civilization: Writing, Remembrance, and Political Imagination* (2011) in the realm transforming “collective memory” into “cultural memory”. Assmann comprehends the domain of “collective memory” as “communicative” and “cultural memory”, since according to him ‘ *a person’s memory forms itself through his or her participation in communicative processes … We only remember what we communicate and what we can locate in the frame of the collective memory*’ ( Assmann, 2011, p. 23). However, channelizing cultural memory in a society takes ‘ *institutionalized mnemotechnics*’ ( Assmann, 2011, p. 37–39) which is aided with symbolic figures so that memory can be attached to it. This multifaceted domain of cultural memory can only sustain itself through people and architecture which can possibly sing the past to the present generation and pass the cultural heritage. Therefore, Assmann stresses the fact that cultural memory ‘ *always has its specialists … cultural memory is disembodied. In order to function as memory, however, its symbolic forms must not only be preserved but also circulated and re-embodied in a society*’ ( Assmann, 2008c, pp. 110–111). These have been well elucidated by Aleida Assmann, a German professor of English and Literary Studies aligning media with cultural memory as writing, image, body and places. Prospects of cultural memory interplay can only be functional when the permeation of images, historical facts and figures has been synthesized and transferred to the successive generation to which Assmann proposed as “Memory Figures”. These memory figures are characterised with three special features: “a concrete relationship to time and place”, “a concrete relationship to a group” and an “independent capacity for reconstruction” ( Assmann, 2011, p. 24). These memory figures are a theorisation of the complex media structure that entails the process of identity formation in the group that in turn becomes collective memory, which serves as a proponent of cultural memory as well. Every event projected by memory figures, condenses in the individuals’ consciousness, and gets sedimented in the culture. As multiple implications of the same narrative on people leave an imprint on their collective memory, thus forming cultural memory.

## Research Methodology

While previous studies have extensively scrutinized the novel from feminist, theological and historiographic perceptive ( Kuznetski, 2021; Van Dam and Polak, 2021; Sabo, 2022), more research is needed to address the tangent of cultural memory in the formation of theocratic state which ultimately leads to dystopia. Hence, this article examines Atwoods’ *The Testaments* on the grounds of memory media and figures by Assmanns. The research writing follows a qualitative approach to disseminate the data from the novels through textual analysis. For secondary literature Assmanns’ *Cultural Memory and Early Civilization: Writing, Remembrance, and Political Imagination* (2011) has been used for the study of memory media and figures. The study specifically scrutinizes dystopian fiction as more dearly connected to cultural memory as the sole aim of dystopian fiction is to create a totalitarian society with every citizen having identical motifs and memories, so much so that the distinction between myth and history vanishes ( Assmann, 2008c, p. 113). Mythology and history are two distinct yet interrelated fields of studies therefore using one as the substitute for the other doesn’t make a credible story for a generation to follow. In *The Handmaid’s Tale* excerpts from the Bible are cited as a closure for all interpretive possibilities. This creates a theocratic regime in the sequel, *The Testaments*, where individuals’ memories are tinted with limited accessible knowledge, ensuing a generation with a cultural memory of “mis- communicated” theocracy, as the only governing dictum.

## Architecture

With the cultural memory domain expanding its wings to newer and fresher dimensions, Aleida Assmann suggests architecture as one media for cultural memory. *The Testaments* being based on a theocratic regime *‘endow texts, persons, artifacts, and monuments with a sanctified status to set them off from the rest as charged with the highest meaning and value’* ( Assmann, 2008a, p. 100). Initiating from a former secondary school, the Rachael and Leah Centre, aka Red Centre, where Handmaid’s got their instructions of duty, which acted as headquarter for patriarchal teachings to obliterate the women/handmaids and strip them of their former life’s memories. Repeated chanting in a Re-Education centre such as ‘ *Oh God, King of the universe, thank you for not creating me a man. Oh God, obliterate me. Make me fruitful. Mortify my flesh, that I may be multiplied. Let me be fulfilled …*’ became a flourishing ground for crumbling individual memories and forming new cultural memories ( Atwood, 2012, p. 194). Initially, Offred tried to resist the patriarchal ideology of Gilead, however in the end, when she was on the brink of losing hope, these words became the voice of her soul rather than a mundane chanting. This repetition of routines in sync and ritualistic chanting helps the group to solidify their collective memories as Jan Assmann suggests ‘ *the role of external symbols becomes even more important, because groups which, of course, do not “have” a memory tend to “make” themselves one by means of things meant as reminders such as monuments, museums, libraries, archives, and other mnemonic institutions*’ ( Assmann, 2008c, p. 111) *.*

Gilead itself was an area of discarded yet unavoidable history of the Puritans, which served as a foregrounded mythical landscape where manipulating Biblical text and memory accentuates the individual identities. As Atwood mentioned in the introduction of the text, ‘ *the Republic of Gilead is built on a foundation of the seventeenth-century Puritan roots that have always lain beneath the modern-day America we thought we knew*’ ( Atwood, 2012, p. 12) *.* Yet to reaffirm this biblical past a schematic narrative needed to be played out in front of the people so that they became prone to reaffirm the values as a cultural memory. James V. Wertsch, in his seminal work *The Narrative Organization of Collective Memory* differentiates between this specific narrative and schematic narrative template where constant revision of the official history overcomes the specific narrative and becomes schematic narration ( Lužný, 2017, p. 200). Thereby, in dystopian literature they carve their own narrative template by using forgotten and fragmented cultural memories in combinations to achieve a linear narrative of their own choice. And architecture such as the Rachael and Leah Re-Education centre or the office of eyes serves as a narrative setting ground. Although Gilead still had to bear some monuments which forebear the liberal past it tried to unsee, we got a glimpse of these monuments from Offred when she mentions that the monuments of recent history do not go well with Gilead authorities: ‘ *The old gravestones are still there, weathered, eroding, with their skulls and crossed bones, memento mori, their dough-faced angels, their winged hourglasses to remind us of the passing of mortal time, and, from a later century, their urns and willow trees, for mourning. They haven’t fiddled with the gravestones, or the church either. It’s only the more recent history that offends them*’ ( Atwood, 2012, p. 42) *.* These monuments carry with them the grave danger of igniting individual memories relating to it, the memories which Gilead’s system tries to overshadow with new collective memories.

Harald Welzer in his chapter *History and Development of the Concept of “Communicative Memory”* addressed the deliberate attempt of the politicians to temper with the architecture *‘historicism and postmodern architecture have shown, this social memory may well be a result of intentional memory politics: Architects and city planners not infrequently set out to accentuate a particular construction of a city’s history, by emphasizing certain historical elements and destroying others’* ( Welzer, 2008, p. 288) *.* Gilead too reconstructed the libraries that in due course of time in *The Testaments* serve no purpose to the citizens especially women as they are not even taught to read. Hence, the tampering with the architecture of the library made it no place of relevance in the cultural memory: ‘ *The Eyes hold sway in a former grand library. It now shelters no books but their own, the original contents having been either burned or, if valuable, added to the private collections of various sticky-fingered Commanders*’ ( Atwood, 2012, p. 56). The handmaid’s and other women of Gilead except for the aunts were deprived of the power to read and write instead they were taught that ‘ *pen is envy*’ ( Atwood, 1996, p. 196). ‘ *A large Eye with a real crystal in the pupil is centred on the door’* (Atwood, 2020, p. 71) took the place of an earlier book storage unit and research cubicles. This act of establishing the office of Eyes in the former library passes a message of redundancy and insignificance of books for the women of Gilead. Whereas, men/commanders were facilitated with a study of their own in their houses where they spend their hours in brooding. While the “Eyes” (security of Gilead) was given a lidless shape to affirm the fact in the citizens that ‘ *the lidless Eye of God, they never sleep*’ ( Atwood, 2019, p. 148) *.*

Architectural surveillance was forced on every citizen of Gilead, supposedly more on women/handmaids, where limiting the products and writings in their room was an agenda to limit their thinking capacity, thereby imposing artificial collective memories on them: *‘I go to the window and sit on the window seat, which is too narrow for comfort. There’s a hard little cushion on it, with a petit-point cover: FAITH … It’s the only thing they’ve given me to read* *’* ( Atwood, 2012, p. 62) *.* The architecture of Offred’s and all the handmaids’ rooms was such that both spectacle and vision for future was suppressed, so that only the training in the Re-Education centre found their place in her mind and solidified its existence as a cultural memory with time. This restrictive architecture of Gilead was contrasted by the building in Canada named “Religious Society of Friends (Quakers)”, a place where Offred’s daughter and Gilead’s most sought-after baby, “Baby Nichole” got shelter. The building was given the name Quakers to show a sharp contrast to Gilead’s ideology as Quakers rejected all elaborate religious ceremonies and believed in the spiritual equality of men and women.

In *The Testaments* these handmaids had passed a generation where their kids, given to the foster parents, had seen Gilead as the only world structure. A structure which is concrete and the architecture bearing it is impenetrable. Even the “Vidala school” has four pictures of *‘Aunt Elizabeth and Aunt Helena, then Aunt Lydia, then Aunt Vidala. Baby Nicole and Aunt Lydia had gold frames, whereas the other three only had silver frames’* ( Atwood, 2019) *.* These pictures of aunts gave the memory of piousness and duty as the ultimate goal of women’s life. Although these girls are taught petit point embroidery for being wives in future, still these pictures in school forms an imprint in their mind following a cultural memory. In contrast to this generation who were born and raised in Gilead there are Aunts, who still have remnants of individual memories left in them, from the time when they were *‘spoiled of too much choice’.* Surprisingly enough, the speaker of the later line Aunt Lydia in *The Testaments*, single handedly initiated a war, and to muster up the courage used her individual memories over those enforced Gileadean cultural imprints. The architecture of Gilead provided the original site for this ideal of precision where everything is geared to order, purity, and harmony (93). In contrast to this when Nichole was studying in Canada, she described her school as, “the school I went to was called the Wyle School. It was named after Florence Wyle, a sculptor of olden times whose picture was in the main entrance hall. The school was supposed to encourage creativity, said Melanie, and understanding democratic freedom and thinking for yourself, said Neil” ( Atwood, 2019, p. 49). Whereas the architecture in Gilead with high walls and stone red buildings was permeated by security measures that indicate a profound awareness of danger – the fear of profanation ( Assmann, 2011, p. 159). Architecture remained the backbone for the people who were capable of reminiscence, such as Aunt Lydia, *‘I am pleased to relate that no one has erased the murals on either side of this building’s interior staircase: since they depict dead soldiers, angels, and wreaths of victory, they are pious enough to have been deemed acceptable, although the flag of the erstwhile United States of America in the right-hand one has been painted over with that of Gilead’* ( Atwood, 2019, p. 56). This in turn indicates the degree of sanctity that must be preserved and that evidently goes far beyond the need for inaccessibility and secrecy that was traditionally associated with holy places ( Assmann, 2011, p. 159).

## Identical Grouping

The cultivation of memory within a society is crucial for defining its temporal and cultural identity. Memory culture extends beyond mere recollection of historical events; it involves creating a collective identity and socially constructing meaning. In order to instill new societal values, the dystopian society of Gilead divided its population into distinct groups based on their functions. The Son of Jacob were the original men who annihilated the United States Congress, overthrowing the country and erecting Gilead in its place. Atwood skilfully depicts the power dynamics within this society, where a few influential men maintain control over the new theocratic state as the Commanders of the Faithful, while lower-ranking men are categorized as Angels, Guardians of the Faith, and Eyes of the God. Angels serve as frontline soldiers, Guardians handle routine policing and menial labor, and Eyes function as the secret intelligence unit of the Republic of Gilead, akin to spies in the real world.

In contrast, Gileadean society segregates women into six categories according to their usefulness towards the society: Aunts, Wives, Marthas, Handmaids, Econowives, and Unwomen. This segregation of women on the basis of their fertility paves the way for further indoctrination of shared memory in the society. Their routines and working patterns were forked in a way that restricts their natural thinking pattern and hence forces them to think like the group they are in: *“Women are either pampered but powerless trophy wives, humble servants known as Marthas or fertile breeding stock”* ( Bianculli, 2017). The groupings are also segregated by coloring. Aunts are given brownish-green robes to project their status of military authority and power of indoctrination. Whereas, wives are donned with lavish blue dress depicting royalty, Marthas wear dull green for household chores and Handmaid’s are the only women allowed to wear vibrant red which depicts their fertility, the most prized possession of Gilead.

This stratification of hierarchy enforced by the ruling elites in Gilead reflects Assmann’s concept of ‘canonization’ in cultural memory, where specific narratives and identities are elevated while others are suppressed or erased. In ‘The Handmaid’s Tale,’ the conditioning of Handmaids goes beyond mere obedience, as these women of the first generation contribute to the formation of a lasting cultural memory for the second generation. The Commanders and Aunts control the religious narratives that define Gilead’s temporal and cultural identity. Memory in Gilead extends beyond historical events; it involves the deliberate shaping of a collective identity through selective narratives and social structures. Atwood’s *The Testaments* features three narrators: Aunt Lydia, Agnes Jemima and Daisy.

The novel opens with Agnes Jemima aka Aunt Victoria’s testimony (369A) where she narrates how Gilead is actively attempting to erase its history, which is marred by corruption, violence and wrongdoings. In an aim to create “a clean space for the *morally pure generation*” ( Atwood, 2019, p. 4), since the beginning of the novel, Atwood highlights the totalitarian society’s deliberate efforts to whitewash its past and enforce moral purity in the second generation exclusively to women. This implicitly reflects the involvement of oppressive propaganda, tactics and suppression of individual freedom, which operates under the guise of security, where little girls from second generation are taught to act, dress and think in certain way, since “urges of men were terrible things” ( Atwood, 2019, p. 9). This hierarchy forming identical groupings were eminent even in children, since they follow dress codes in accordance to their parents’ socioeconomic status.

“ *The pink, the white and the plum dresses were the rule for the special girls like us. Ordinary girls from Econofamilies were the same thing, all the time, those ugly, multicolour striped and grey cloaks like the clothes of their mothers.”* (11)

Agnes’s childhood memories are tainted with experiences of oppression and social stratification within the regime. The powers within the regime attempt to normalise imposition of oppressive power structures by associating certain colour codes like “pink, white and plum” with fashion and privilege, while labelling the girls who wear these colours as “special girls”. On the contrary, Econogirls are dehumanized based on their “ugly, multicolour striped and grey cloaks”. Their clothing represents their lack of agency and further heightening a stark contrast of power in their cultural memory within the social fabric. The regime’s efforts to reinforce and perpetuate social hierarchies through visual markers situates the memory in motion. The memory becomes mobile *in the sense that it is created through exchanges between different subjectivities* ( Passerini, 2021, p. 2) *.* Agnes’ memory acts as a historical agency, which plays a formative yet behind the scene role in the construction of new monolithic history.

Homogeneity of the experience was curated to such an extent that every small detail starting from uniform to behaviour was enforcing some sort of collective memory amongst societal structure. Assmann explains when mimetic memory, memory of things and communicative memory merge, they form what he called ‘cultural memory’ ( Assmann, 2011, p. 6). The prime function of cultural memory is to hand down the cultural meaning across generations. In the novel, these meanings are handed down through rituals, ceremonies and virtual representations. The dollhouse provided to children, especially girls reflect a meticulously crafted replica of Gileadean society.

“ *We had all the dolls for the doll house that you might need a mother doll in a blue dress of Commanders’ Wives, a little girl doll with three dresses, pink, white, and plum, just like mine, three Marthas stall in dull green with aprons, a Guardian of the Faith with the cap to drive the car and mow the lawn, two Angels to stand at the gate with their miniature plastic guns, so nobody could get in and hurt us, and a father doll in a crisps Commander’s uniform.*” ( Atwood, 2019, p. 14-15)

The doll house seamlessly reflects all three aspects of cultural memory, as it reiterates the predefined roles of each category, simultaneously acting as the tangible representation of the memory and lastly it communicates the regime’s rules and ideologies to the newer generation.

Throughout Agnes’s narration in the novel, the doll house appeared persistently, accentuating its importance in her memories and understanding of Gilead’s operations. She exhibited her entire understanding of Gilead’s working and its rigid environment to readers through the medium of dollhouse. The subtle hints of rebelling against the taut predefined roles are seen, when she refused to become a wife to a commander despite being trained her entire life for this purpose. To intensify the dramatization of anger felt by Agnes and to make her agency visible, Atwood again utilized the doll house as a powerful symbol.

*There was the Wife doll, sitting at the dining table; there were little girls, behaving themselves; there were the Marthas in the kitchen, making bread; there was a Commander, safely logged into his study. After Paul had left, I plugged the Wife doll out of her chair and threw her across the room* (157).

Agnes willingly rejects the virtues instilled in every vein of Gilead: *obedience, subservience, and docility* (291). She ascends within the regime’s power hierarchy by becoming an Aunt, where she confronts the darker realities of the state. While, other citizens believed that Aunts held the power and liberty, Aunt Lydia’s narration highlights how they were forced by Son of Jacobs to unquestionably follow their commands, confining their existence to the ‘brown sackcloth robe’. The robe that defines their place and identity in Gilead bestows upon them a sense of purity and authority both at the same time. Aling with Assmann’s notion of ‘canonization’, which highlights that *canonical text requires the presence of a third party – the interpreter – to mediate between the text and the reader/listener, and to clarify the meaning hidden within the words* ( Assmann, 2011, p. 79). Atwood grants the privilege of interpreting selected religious excerpts to Aunts. In *The Testaments*, this narrative interpretation has been majorly done by Aunt Lydia who’s the first generation of aunts, *their task is both to embody and to spread its authority and its revealed truth* (80). The formation of identical memories was done to the extent of food where “diet is monotonous, and a little variation is welcome, even if only a variation in the colour” ( Atwood, 2019, p. 33). Aunts played a major role in transmitting the cultural memory across generations, which was besmirched by oppression disguised as security. However, Aunts themselves were uniformly grouped by the Sons of Jacob, leaving them with no alternative but violence in order to survive, resulting in their collective memory being burdened with the weight of guilt.

## Language and Memory

Historically, language and memory have been studied independently. Ignoring the influence of language over memory, scholars created a neuro-linguistic gap between the past and present. Identical to language, memory also helps in the formulation of one’s identity, by socially constructing meanings over time. Culture in memory, primarily dangles on the countless links with the past. Jan Assmann has bridged the gap between the two, by revealing the working of communicative memory, which operates on *“circular or feedback interplay between interior and exterior”* ( Assmann, 2011, p. 6). Further, it contributes to the formulation of one’s individual memory and consciousness through the continuous practices carried out within the culture, that implicitly affects the cultural memory of the state. Thus, he states that *“study of cultural memory therefore focuses on such processes of transformation and enhancement, examining the decisive changes within the connective structure of a given society.” (10)*

One of the significant decisive changes that occurred in Atwood’s duology is ‘language’, which contributed to the cultural shift of the state, a shift from the democratic America to the theocratic Republic of Gilead. The new government fixes their eyes on tomorrow while *“preserving yesterday from oblivion by grasping it through memory*” (Assmann, 17). The officials of Gilead keep referring to biblical text and language to bring the past into people’s conscious memory.

Assmann presumes two things which help in producing the cultural memory through the communicative power of language:

*“There has to be some sort of record or documentation of the past one trying to refer to.*

*This record must indicate a distinguishing feature from today.” (18)*

This can be illustrated with the example of Atwood’s *The Testaments*: where books were kept decoratively in the house library and women were forbidden to read them. Similarly, in its prequel, the narrator, Offred witnesses the recitation of some excerpts from the Bible and also few foreign words like “ *m’aidez*”, “ *café au lait*, *nolite te bastardes carborundorum*”, “ *sum es est”, “sum us estis sunt*”, et cetera (which were antecedent to the current used language).

Furthermore, Atwood established what Assmann called ‘canon’ in the former novel, which continued until the end of *The Testaments.* ‘Canon’ *stands for a particular case – ‘that of a principle, norm, or value that is far more binding than a unique code such as a language’s grammatical rules’* ( Assmann, 2011, p. 98). Canon emphasises using *"highly normative grammar or ideologically conditioned form of language"* ( Assmann, 2011, p. 98). The second generation now adapts to the rules and norms set by their predecessors in *The Handmaid’s Tale.* The official language of Gilead aspires to repudiate and prohibit the former speech with biblical discourse ( Kouhestani, 2013 p. 661). Gilead is still operating with the same lexical units, and young girls are conditioned to speak in the more rigid biblical language and stick to the teaching of their elders: “Just learn your lessons and trust your elders to do what is best, and everything will unfold as it should” (Atwood, 10). Words associate themselves with various meanings, but Biblical teaching in Gilead restricts the meaning of the lexeme towards a particular direction. Gilead uses language as a medium to memory in the psyche of Gileadean citizens which normalizes the subjugated role of women as ‘birth vessels’.

Assmann categorized the derivation of meanings in four categories, namely (1) measure, ruler, criterion (2) model (3) rules, norm and (4) table and list.

This *‘measure, ruler, criterion’* category allows the authorities to deduce the sections to a minor part proportional to the whole and a functional unit. These parts of the excerpts in isolation convey different meanings opposing or diverting it from the true sense. Similar practices are carried out by the theocratic state, where some selected sections are repeatedly taught to the woman. In the words of Assmann, Aunts use the *“faithful-to-word-and-meaning”* ( Assmann, 2011) formula for forking context from the text and for generating meaning. This separation of context from the selected extracts majorly contributes to the diversion of meaning, contributing to the justification of Gilead’s authoritarian rule. To maintain their rule, the state prohibited women from reading the whole bible, other documentation and world literature, locking it up in the Hildegard Library since it would uncover their truth ( *‘rule, norms’*): “The precious Bloodlines Genealogical Archives kept so meticulously by the senior Aunts, the Bibles, the theological discourses, the dangerous works of world literature—all were behind that locked door” (Atwood, 300). Henceforth, rules are created to blur the context of the Bible and to create a distinct record of Gileadean context to Biblical excerpts.

The ‘ritual continuity’ of practices in Gilead helps the governing bodies hold power over women through birth ceremonies and other events (like birthdays, hymns) where women are repeatedly taking part in and handmaids learning similar Biblical phrases daily, including “Uplift the Lowly” and “Blessed Be the Fruit”. Consequently, it manifests itself as a ‘created reality’ or ‘distorted Biblical truth’ in the psyche, creating a long-term memory. This recurring interplay of experiences and concepts transpose the original memory figures, making ‘captured children and women’ forget their original individual memory: *“We can’t remember what it is that we’ve forgotten. That we have been made to forget. That we’ve had to forget, in order to pretend to live here in any normal way”* (330).

The first-generation of the Handmaids act as a *‘model’* for the second generation. A *model* can be defined as the “canon of conduct” (Assmann, 93). Growing up seeing the restricted and divided roles of women in the household, the second generation normalized and accepted the subjugated position of women, whom Gilead prepares either to marry a commander or to become a handmaid. The Latin language, which had almost vanished from the documentation in Agnes and Baby Nicole’s generation, left behind a few phrases such as *“Per Ardua Cum Estrus”*, *“memento mori*”. In contrast, the former generation which was aware of the many foreign languages such as Latin, Italian and French but in a rather controlled and restricted environment results in the oblivious state of Agnes about the language: *“‘What is Latin?’ Becka said it was a language of long ago that nobody spoke anymore, but people wrote mottoes in it”* (Atwood, 289).

Apart from Latin, archaic words and biblical jargons also dominate Gilead’s language system starting from the name Gilead itself which is a place in the Bible, which symbolises the hope for restoring that had been destroyed (98). In the state, very few officials have access to the Bible due to its controversial usage. When Agnes gets access to her Bible, she learns that *“It does not say what they say it says”* (302). Agnes realizes that the excerpts she had been reading and memorizing since childhood were the pericopes for their ideological conditioning. The excerpts are separated from their context so that Gilead can use them to brainwash women and create identical individual memories, contributing to collective cultural memory formation. From here, Gilead started falling apart, and the collective memory made by Gilead also started fading. Although, no one remembers their past and language, they witness some glimpses of flashes from their past. As the theocratic state dissolved completely, Agnes was astonished that she knew only the restricted vocabulary, which limits her language and memory consequently moving ahead with Nicole would be difficult: “ *“*Gilead,” said Nicole, “is not where we’re going. We’ve got two minutes to join our buddy outside. So suck it up.” “Pardon?” Sometimes I could not make out what my sister was saying…“It means ‘be brave,” she said *. We are going to a place where she will understand the language, I thought. And I will not.* (364). This case of language distinction on the part of Agnes and Nicole can be well elucidated by the Sapir-Whorf Hypothesis where it states that your language shapes your thinking and social reality to a great extent. Therefore, a shift in language makes a shift in the cultural identity and hence memory of a person.

## Conclusion

Memory plays a prominent role in creating one’s identity hence manipulating one’s memory can have a direct impact on their identity. Similar patterns of cultural memory formation or in other words individual memory distortion at macro scale was evident in the text of *The Testaments.* The story is narrated from three perspectives: second generation’s perspective (Agnes), powerful elite’s perspective (Aunt Lydia) and outsider’s perspective (Baby Nicole). Each narration reflects a different perspective on the power dynamics within Gileadean society. Aunt Lydia’s perspective offers insight to how power is exercised and maintained by powerful elites within Gilead. She narrates the story from the viewpoint of someone who actively participates in and manipulates the regime’s ideology to consolidate her own power and influence to stay alive. Agnes Jemima aka Aunt Victoria’s perspectives uncover her experiences as a normal citizen in the society, her narration reveals the day-to-day experiences and challenges faced by individuals trying to navigate the oppressive society imposed by the regime. Daisy aka Baby Nichole aka Jade offers an outsider’s perception of Gilead, having been rescued and raised outside of Gilead. Daisy’s perspective provides a stark contrast between life within and outside the totalitarian regime.

The second generation is born within the regime of Gilead. They do not witness the transition from a democratic state to the totalitarian society, where women are oppressed and assigned the roles based on their fertility. Even children captured or born during the conversion of state have blurred memory or no memory about the free world they used to live in. Agnes Jemima, being one of the narrators, struggles throughout the novel to recall her childhood memories and uncover the truth about her birth. When she discovers that Tabitha is not her real mother, her entire belief system begins to shatter. Therefore, Jan Assmanns’ Memory Figures proved to be an apt theoretical base for Atwoods’ work. Architecture and language are the two subtle mediums used to communicate the frames of collective memory of the younger generation in *The Testaments.* The prominence of cultural imprints are visible in the bipolar attitudes of Daisy aka Baby Nicole and Agnes as one is raised in Canada and the other in Gilead. Identical grouping is one such tactic applied by the theocratic regime to create a hierarchical setup for generations to follow. Language plays a dominant role in canonization of memory from naming Gilead to limiting library access to women, thereby setting selective narrative.

## Data Availability

All data underlying the results are available as part of the article, and no additional source data are required.

## References

[ref1] Assmann A : Cultural Working Memory: The Canon. Noơnning AEA , editor. *Cultural Memory Studies: An International and Interdisciplinary Handbook.* Walter de Gruyter;2008a; pp.100–107.

[ref2] Assmann A : Transformations between history and memory. *Social Research: An International Quaterly.* 2008b;75(1):49–72. 10.1353/sor.2008.0038 Reference Source

[ref3] Assmann J : Communicative and Cultural Memory. Erll A Nünning A , editors. *Cultural memory studies: an international and interdisciplinary handbook.* Vol.2008. Walter de Gruyter;2008c; pp.110–111. 10.1515/9783110207262

[ref4] Assmann J : *Cultural memory and early civilization: Writing, remembrance, and political imagination.* 2011th ed. Cambridge University Press;2011. 10.1017/CB0O9780511996306

[ref5] Atwood M : *The Handmaid’s Tale.* Vintage Books;1996.

[ref6] Atwood M : *The Handmaid’s Tale.* 1st ed. Vintage Books;2012.

[ref7] Atwood M : *The Testaments.* 1st ed.Vol.2019. London: Chatto and Windus;2019.

[ref8] Bianculli D : Hulu’s ‘Handmaid’s Tale’ Delivers a Timely and Feminist Message. *NPR.* 26 Apr. 2017. Reference Source

[ref9] Funkenstein A : Collective Memory and Historical Consciousness. *Hist. Mem.* 1989;1(1):5–26. Indiana University Press. Reference Source

[ref10] Halbwachs M : *ON COLLECTIVE MEMORY.* COSER LA , editor. The University of Chicago Press;1992. 10.1016/j.lisr.2016.08.010

[ref11] Karuny: Lev Vygotsky. *Pedagogy4Change.* 28 July 2022. Reference Source

[ref12] Kouhestani M : Disciplining the Body: Power and Language in Margaret Atwood’s Dystopian Novel The Handmaid’s Tale. *J. Educ. Soc. Res.* 2013;3:610. 10.5901/jesr.2013.v3n7p610

[ref18] Kuznetski J : Disempowerment and bodily agency in Margaret Atwood’s The Testaments and the Handmaid’s Tale TV series. *The European Legacy/the European Legacy, Toward New Paradigms.* Mar. 2021;26(3–4):287–302. 10.1080/10848770.2021.1898108

[ref13] Lužný D : Sect as a Threat- Cultural Memory and the Image of Sects. *Politics Relig. Ideol.* 2017;18(2):198–216. 10.1080/21567689.2017.1330685

[ref14] Nora P : *Between Memory and History: Les Lieux de Memoire.* University of California Press;2011; vol.26:7–24.

[ref15] Olick JK Robbins J : Social Memory Studies: From ‘Collective Memory’ to the Historical Sociology of Mnemonic Practices. *Annu. Rev. Sociol.* 1998;24:105–140. Annual Reviews. 10.1146/annurev.soc.24.1.105 Reference Source

[ref19] Passerini L : The Mobility of Memory in the Context of Intersubjectivity. *The Mobility of Memory: Migrations and Diasporas across European Borders.* Berghahn Books;2021. 10.1515/9781789202342

[ref16] Quan Z : Cultural Memory and Ethnic Identity Construction in Toni Morrison’s A Mercy. *J. Black Stud.* Sept. 2019;50(6):555–568. 10.1177/0021934719861268

[ref20] Sabo PJ : Testifying Bodies: The Bible and Margaret Atwood’s the Testaments. *Journal of Feminist Studies in Religion.* Apr. 2022;38(1);131–147. 10.2979/jfemistudreli.38.1.24

[ref21] Van Dam D Polak S : Owning Gilead: Franchising Feminism Through Margaret Atwood’s the Handmaid’s Tale and the Testaments. *European Journal of English Studies.* May 2021;25(2):172–189. 10.1080/13825577.2021.1950362

[ref17] Welzer H : History and Development of the Concept of “Communicative Memory.” Noơnning AEA , editor. *Cultural Memory Studies: An International and Interdisciplinary Handbook.* Vol.8.2008; pp.285.

