# Supplementary material for: “Gilead is within you”: A Theocratic Narrative Setting of Cultural memory in Atwood’s The Testaments
Source: F1000Res. 2024 May 28;12:475. Originally published 2023 May 9. [Version 2] doi: 10.12688/f1000research.132487.2 (PMC10514572; doi:10.12688/f1000research.132487.2)
Supplement: Peer review history

# Reviewer report: Reviewer response for version 2

**DOI:** 10.5256/f1000research.166536.r283771

**Published:** 2024-06-05

**Authors:** Enrique Meléndez Galán

**Affiliations:** 1 Universidad de Oviedo, Oviedo, Spain

More additional content is not necessary.

Is the work clearly and accurately presented and does it cite the current literature?

Partly

If applicable, is the statistical analysis and its interpretation appropriate?

Not applicable

Are all the source data underlying the results available to ensure full reproducibility?

No source data required

Is the study design appropriate and is the work technically sound?

Yes

Are the conclusions drawn adequately supported by the results?

Yes

Are sufficient details of methods and analysis provided to allow replication by others?

Partly

Reviewer Expertise:

Visual Culture, Speculative Fiction, Contemporary Art, Transmission of Knowledge

I confirm that I have read this submission and believe that I have an appropriate level of expertise to confirm that it is of an acceptable scientific standard.

# Reviewer report: Reviewer response for version 1

**DOI:** 10.5256/f1000research.145411.r196129

**Published:** 2023-09-21

**Authors:** Lizzie Seal

**Affiliations:** 1 Professor of Criminology, University of Sussex, Brighton, England, UK

This article deploys theories of collective and cultural memory to analyse Atwood’s portrayal of a theocratic dystopia in The Testaments. It applies these theories thoroughly and effectively to examine how the dystopian regime works and reproduces itself. The sections on architecture and language are fruitful. I have some comments and suggestions regarding how the article could be improved.

1. The article would benefit from a more extensive summary of the novel to ground the discussion.
2. The authors need to clarify which body or bodies of academic literature they are seeking to contribute to.
3. Previous literature on The Handmaid’s Tale and The Testaments should be incorporated into the literature review, as well as literary criticism on collective memory and dystopia more widely.
4. The identity separation section is very short - I recommend removing it.
5. The conclusion is too brief and needs expanding. The authors can fully outline their contribution here, taking existing literature into account.
6. The article needs a thorough proof read for grammar and syntax (particularly for misplaced commas). Attention is also needed to errors in word choice - for example, it should be ‘democratic America’, not ‘demographic’.

Is the work clearly and accurately presented and does it cite the current literature?

Partly

If applicable, is the statistical analysis and its interpretation appropriate?

Not applicable

Are all the source data underlying the results available to ensure full reproducibility?

No source data required

Is the study design appropriate and is the work technically sound?

Partly

Are the conclusions drawn adequately supported by the results?

Yes

Are sufficient details of methods and analysis provided to allow replication by others?

Yes

Reviewer Expertise:

Historical criminology; cultural criminology

I confirm that I have read this submission and believe that I have an appropriate level of expertise to confirm that it is of an acceptable scientific standard, however I have significant reservations, as outlined above.

# Reviewer report: Reviewer response for version 1

**DOI:** 10.5256/f1000research.145411.r193172

**Published:** 2023-08-30

**Authors:** Enrique Meléndez Galán

**Affiliations:** 1 Universidad de Oviedo, Oviedo, Spain

Summary: The paper examines Margaret Atwood’s *The Testaments* by analyzing Memory Figures (architecture and time, identical groupings, and language). The study concludes that manipulating one’s memories can build a new identity, in this case, for a religious dystopia. On this way, using memory figures is helping to analyze different attitudes of two of the main characters.

 Strengths:

- Original point of view and accurate methodology.
- Clear correlation between characters, book descriptions and Memory Figures.

Weaknesses:

- Lack of discussion on identical grouping, doing unbalanced this part of the paper in comparison with the other sections.
- Unlimited field of work, analyzing examples from Atwood’s *The Testaments *and, also, *The Handmaid’s Tale *when the title of the paper is focused just in *The Testaments.*

Suggestions for Changes:

1. **Broaden the Scope:** Introduce a comparative analysis by considering, from the beginning of the paper, references from both of the novels written by Atwood about Gilead to provide a more comprehensive understanding of the figures. The authors are not being limited to *The Testaments, *and consequently, it must be clear from the beginning.
2. **Address Alternative Factors:** Discuss the potential influence of other factors, specially in identical grouping, such as patriarchy, different roles of the women in the society, or different categories for the men to offer a well-rounded perspective and balance this part of the paper.
3. **Include a context quoting the Tv Serie:** Extend the context beyond the novels to capture broader field of work on visual and cultural studies and helping to understand the importance of this novel after being a TV Series.
4. **Propose Analysis Strategies:** Offer recommendations for future analysis on this way to replicate the use of Memory Figures on other dystopian novels.

By implementing these changes, the paper can offer a more complete view of the importance of the role played by memory figures on the constructions of the characters and provide a well-rounded analysis of the world building according not only to *The Testaments* but to *The Handmaid’s Tale *also.

 On the other hand, it would be interesting to correct this issues:

1. At the beginning of the article is written “Daily” instead of “Daisy”. Review.
2. “Memory Figures” is once written with capital letters and other times without capital letters. Unify.
3. Since authors are using two sources with the same surname, it would be good for a better understanding, if editors approve, including the second surname in the quote or the initial of the name to clarify the source. Review.

Is the work clearly and accurately presented and does it cite the current literature?

Partly

If applicable, is the statistical analysis and its interpretation appropriate?

Not applicable

Are all the source data underlying the results available to ensure full reproducibility?

No source data required

Is the study design appropriate and is the work technically sound?

Yes

Are the conclusions drawn adequately supported by the results?

Yes

Are sufficient details of methods and analysis provided to allow replication by others?

Partly

Reviewer Expertise:

Visual Culture, Speculative Fiction, Contemporary Art, Transmission of Knowledge

I confirm that I have read this submission and believe that I have an appropriate level of expertise to confirm that it is of an acceptable scientific standard, however I have significant reservations, as outlined above.

# Reviewer report: Reviewer response for version 1

**DOI:** 10.5256/f1000research.145411.r184137

**Published:** 2023-08-21

**Authors:** Teresa Tudu

**Affiliations:** 1 Department of English, J N College, Ranchi, Jharkhand, India

In this article, the authors explored how certain acts like identical grouping and language control shape the cultural memory in Margaret Atwood’s “The Testaments”. As a methodological framework, the article uses Jan Assmann’s concept of Memory Figures to highlight the formation of collective memory, which further imprints itself as cultural memory in the minds of characters over time in the novel. The article also provides a clear and concise literature review to track the development of the concept of memory over the years. Despite falling under the domain of memory studies, this research also highlights the well-rounded underline pattern of creating cultural memory through language, literature and identical grouping in any totalitarian society.

 Through a meticulous analysis of the impact of language on the formation of cultural memory and identity, the article makes a valuable contribution to the broader discourse surrounding social constructs, power dynamics, and the shared awareness of communities, both within the realm of literature and in the tangible world. Further, it enriches the existing body of knowledge surrounding Margaret Atwood’s literary contributions by providing the novel viewpoint on the complex interplay between language, memory, and identity. This, in turn, fosters a more profound understanding and admiration for her storytelling prowess.

 The article exhibits promising potential. However, I would like to kindly offer some suggestions to the authors in order to further enhance this article. Additionally, I am looking forward to receiving a revised version of the manuscript from the author.

1. The authors briefly mention "The Handmaid's Tale" as a precursor to "The Testaments" in the abstract, but the rest of the article lacks a comparative analysis of both novels. It would be beneficial if the authors included a more comprehensive comparative analysis between the two novels in order to further delve into the exploration of cultural memory and identity to highlight the evolution of the two across the novel.
2. The article could benefit from considering other relevant theoretical frameworks or literary criticisms in addition to Jan Assmann's concept of Memory Figures. This would help provide a more comprehensive understanding of the intricate dynamics of cultural memory as portrayed within the novel.
3. Lastly, the conclusion of this article is succinct but it somehow fails to provide a comprehensive synthesis of the findings derived from the analysis. It will be better if authors add an intellectually stimulating conclusion to add more clarity to this research. 

Is the work clearly and accurately presented and does it cite the current literature?

Yes

If applicable, is the statistical analysis and its interpretation appropriate?

Not applicable

Are all the source data underlying the results available to ensure full reproducibility?

Yes

Is the study design appropriate and is the work technically sound?

Yes

Are the conclusions drawn adequately supported by the results?

Yes

Are sufficient details of methods and analysis provided to allow replication by others?

Yes

Reviewer Expertise:

Cultural Studies, Tribal literature

I confirm that I have read this submission and believe that I have an appropriate level of expertise to confirm that it is of an acceptable scientific standard.
